# Improvement of intestinal flora: accompany with the antihypertensive effect of electroacupuncture on stage 1 hypertension

**DOI:** 10.1186/s13020-020-00417-8

**Published:** 2021-01-07

**Authors:** Jun-meng Wang, Ming-xiao Yang, Qiao-feng Wu, Ji Chen, Shu-fang Deng, Lin Chen, Da-neng Wei, Fan-rong Liang

**Affiliations:** 1 Acupuncture and Moxibustion College, Chengdu University of Traditional Chinese Medicine, No.37, Road Shi-Er-Qiao, Jinniu District, Chengdu, 610075 Sichuan China; 2 School of Chinese Medicine, The University of Hong Kong, 10 Sassoon Road, Pokfulam, Hong Kong, SAR China; 3 Department of Chinese Medicine, The University of Hong Kong-Shenzhen Hospital, Hai yuan Road, Futian District, Shenzhen, 518053 Guangdong China; 4 Institute of Acupuncture and Homeostasis Regulation, Chengdu University of Traditional Chinese Medicine, Chengdu, 610075 Sichuan China

**Keywords:** Intestinal flora, Electro-acupuncture, Stage 1 hypertension, Dysbiosis

## Abstract

**Background:**

Increasing evidence have indicated the relationship between intestinal dysbiosis and hypertension. We aimed to evaluate the effect of the electroacupuncture (EA) on intestinal microbiota in patients with stage 1 hypertension.

**Methods:**

93 hypertensive patients and 15 healthy subjects were enrolled in this study. Applying a highly accurate oscillometric device to evaluate the antihypertensive effect of EA. 16S rRNA sequencing was used to profile stool microbial communities from Healthy group, Before treatment (BT) group and After treatment (AT) group, and various multivariate analysis approaches were used to assess diversity, composition and abundance of intestinal microbiota.

**Results:**

In this study, EA significantly decreased the blood pressure (BP) of hypertensive patients. Higher abundance of *Firmicutes* and lower *Bacteroidetes* abundance were observed in the BT group compared to the Healthy group. And EA treatment significantly decreased the *Firmicutes/Bacteroidetes* ratio compared to the BT group. Moreover, at the genus level, there was an increased abundance of *Escherichia-Shigella* in patients with hypertension, while *Blautia* were decreased, and EA reversed these changes.

**Conclusions:**

Our study indicates that EA can effectively lower BP and improve the structure of intestinal microbiota which are correlate with the alteration of blood pressure by electroacupuncture.

*Trial registration:* Clinicaltrial.gov, NCT01701726. Registered 5 October 2012, https://clinicaltrials.gov/ct2/show/study/NCT01701726

## Background

Hypertension is China's leading cause of cardiovascular diseases (CVD), including stroke, heart failure, coronary heart disease [1]. Because of the aging of the population and changes of life style and living environment, hypertension is increasing in prevalence worldwide [2]. Many studies have shown Stage 1 hypertension (BP 140–159 or 90–99 mmHg), also called mild hypertension [3], was significantly associated with a higher CVD risk and mortality across entire adulthood [4–7].Even among young adults, stage 1 hypertension before age 40 years also had significantly higher risk for subsequent cardiovascular disease events compared with those with normal blood pressure before age 40 years [8]. Therefore, early treatment of hypertension and its sequelae can greatly reduce the cardiovascular medical burden. However, current evidence demonstrated that antihypertensive drugs used in the treatment of patients with stage 1 hypertension do not reduce mortality or morbidity but increase the risk of adverse events in RCTs [9, 10]. As one of the nonpharmacologic treatments, acupuncture has been used to relieve abnormal blood pressure (BP) and its typical organ damages or symptoms for several decades. A recent meta-analysis showed that acupuncture could be used for treating hypertension, and it may have the same effects as common medication [11]. Other studies have also indicated that acupuncture is particularly effective to lower blood pressure in prehypertension and stage 1 hypertension [12, 13]. Hence, acupuncture could be a potential therapy for stage 1 hypertension.

Except for heredity and environment, intestinal microbiota has increasingly been recognized as a main risk factor for hypertension. Changes in composition and dysbiosis of intestinal microbiota were observed in multiple hypertensive patients and animals [14]. Researchers have identified some possible hypotheses linking dysbiosis to hypertension through indirect mechanism including the low-grade inflammation [15], increasing of gram-negative bacteria [16], reduction number of short-chain fatty acids (SCFAs) producing bacteria17, 18, generation of trimethylamine N-oxide [19], and so on. However, few studies have explored the effect of acupuncture on the intestinal microbiota in patients with stage 1 hypertension. We therefore used high-throughput sequencing to determine changes in intestinal microbial community structure in patients received EA treatment. Our results provide new leads regarding the pathogenesis and treatment of hypertension.

## Methods

### Participants

A total of 108 subjects were enrolled:93 hypertensive patients and 15 healthy. All patients received EA treatment. Among them, 29 of whom had feces collected before and after treatment. 15 normal people were also collected feces after enrollment. The study protocol was approved by Sichuan Regional Ethical Review committee of the Teaching Hospital of Chengdu University of Traditional Chinese Medicine (2012KL-003) and conformed to the ethical guidelines of the 1975 Declaration of Helsinki. Patients of essential hypertension were included and clear diagnosis according to the seventh report of the joint national committee on prevention, detection, evaluation, and treatment of high blood pressure. Study participants were recruited from the third teaching hospital of Chengdu University of Traditional Chinese Medicine and its surrounding communities. Participants were enrolled if they fulfilled the following criteria: [1] aged between 45 and 75 years; [2] were diagnosed as stage 1 hypertension in the first visit, or used to be diagnosed as stage 1 hypertension in recent 1 year, but without any medication history; [3] without neurological, other cardiovascular, hepatic and renal disease, and other visceral diseases; [4]without any drugs or herbs in at 15 days before the start of the study; [5] did not participate in any other study; [6] agreed to cooperate with researchers in all research procedures after they were introduced this study; and [7] provided with written informed consent. Patients with any of the following conditions were excluded: [1] with hypertension which was secondary to other diseases, such as renal vascular disease, Cushing’s syndrome, hyperadrenocorticism and drug-induced hypertension; [2] had complicated cardiovascular, digestive, respiratory, urinary, blood, nervous, endocrine system and other severe primary diseases and failed to effectively control in clinic; [3] accompanied by epilepsy, sleep apnea hypopnea syndrome, etc.; [4] with psychiatric symptoms such as severe depression or anxiety (SAS ≥ 70, or SDS ≥ 72); [5] pregnant or lactating woman, or woman of reproductive age who was intended to conceive in recent 1 year; [6] with abnormality in laboratory test of blood biochemistry, or with contagious risks, such as HIV virus carrier, or patient with positive HBV superficial antigen; [7] with malignant tumor or other severe consuming diseases, or patient with infections or bleeding disorders; [8] alcoholics or drug abusers, or vegetarians; [9] used to suffer from acute diseases in recent 2 weeks, such as high fever, or gastritis; [10] used to administer any drug that may potentially impaired renal or hepatic function; or [11] undergoing other clinical trials.

### Interventions

After inclusion, patients received 18 sessions of acupuncture treatment (3 times weekly for 6 weeks). Each session lasted 30 min. We chose bilateral acupoints of taichong (LR3), taixi (KI3), renying (ST9), neiguan (PC6) according to traditional Chinese medicine theory and based on review of the literature. Disposable stainless-steel needles were used in acupuncture treatments. Insertion was followed by stimulation performed by lifting and thrusting the needle combined with twirling and rotating the needle sheath to produce the sensation known as deqi [20].In addition, auxiliary acupuncture needles were inserted 2 mm lateral to each acupoint to a depth of 2 mm without manual stimulation. This method could ensure the electrical stimulation working on the local points. The HANS acupoint nerve stimulators (Model LH 200A; HANS Therapy Co) were used after needle insertion. The stimulation was 2 Hz continuous wave; intensity varied from 0.1 to 2.0 mA until patients felt comfortable. Acupuncture was performed by licensed acupuncturists with more than 5 years’ experience.

### 24 h ambulatory blood pressure monitoring/Ambulatory blood pressure monitoring

24 h ambulatory blood pressure recordings were obtained by a highly accurate oscillometric device (TM-2430, A&D Instruments, Japan) within 24 h before acupuncture treatment and the second day of the final treatment respectively. The TM-2430 monitor measures blood pressure automatically, on the same principle as the conventional mercury sphygmomanometer, with a cuff and a microphone. The interval between measurements can be preselected, and BP readings are recorded on a data processor. Blood pressure measurements were taken automatically every 15 min from 8 AM to 10 PM and every 30 min from 10 PM to 8 AM. If a reading were considered faulty by the device, it was programmed to remeasure, which helped to avoid missing a measurement. The data from the device were then downloaded onto a computer for subsequent analysis. Before the study started, the investigators were trained for the appropriate use of the BP measurement device used in the study, and had to demonstrate proficiency according to the manufacturer’s instruction.

### Fecal microbial DNA extraction

A total 73 Stool samples were collected, of which 15 samples were from healthy, 29 samples were collected before acupuncture treatment and 29 samples were collected at the second day of the final treatment. Fecal genomic DNA was isolated from stool samples by using a QIAamp DNA Stool Mini Kit (51504, Qiagen, German) according to the kit protocol with modifications. The quality of extracted genomic DNA was checked by using the 2% agarose gel, and the DNA concentrations were determined with QuantiFluor™-ST Handheld Fluorometer with Blue Channels (Promega, USA). The extracted DNA was stored at − 20 °C.

### Fecal bacterial identification

Bacteria within each sample were identified via 16S rRNA Sequencing. PCR amplification for the V4 hypervariable regions of the bacterial 16S rRNA gene was performed using the 515F forward (5′-GTGCCAGCMGCCGCGGTAA-3′) and 806R reverse (5′-GGACTACHVGGGTWTCTAAT-3′) primers. Nextera XT V2 index kit Sets A and B were used to barcode PCR products and the AMPure XP bead system (Illumina, USA) was used for purification. Barcoded DNA underwent quantification, normalization and pooling to develop sequencing libraries which were then sequenced on the Illumina MiSeq platform (Illumina, USA). The Quantitative insights Into Microbial Ecology (QIIME) v1.9.1 analysis tool was used to join and de-multiplex forward and reverse sequences. Using the Silva reference database, the open reference operational taxonomic unit (OTU) picking method was used for taxonomic assignments with a pairwise identity threshold of 97%. Prior to downstream analysis, the OTU table was rarefied to 3000 sequences/sample with no samples removed in this step. These OTUs were analyzed by global parameters (described below) including determination of diversity, evenness, richness, and relative abundance of the bacteria identified in each sample.

### Statistical analysis

Paired t-test were performed to compare the baseline and post-treatment blood pressure. 16S rRNA gene sequencing data was analyzed on the free online platform of Majorbio Cloud Platform (www.majorbio.com). Chao1, Ace, Simpson and Shannon index were used for comparison of alpha diversity. Principle coordinates analysis (PCoA) (Bray–Curtis dissimilarity index) and partial least squares discriminant analysis (PLS-DA) were used to visualize the change of distribution patterns. The unpaired two-tailed *t*-test was used for comparisons between Healthy and Hypertension. The paired t-test was used for comparisons between BT group and AT group. Spearman’s correlation coefficients were used to assess bivariate relationships. All results with *P* < 0.05 between groups were considered statistically significant.

## Results

### Baseline information

Among 397 patients screened, **101** were enrolled at baseline. 8 patients never received a treatment and lost contact after baseline. A total of 93 patients received 18 sessions of EA treatment. The baseline characteristics and BP-related parameters such as gender, age, nationality, BMI, and other physiological parameters like blood sugar, total cholesterol, RBC, WBC demonstrated no significant difference in the hypertensive patients and healthy control. However, the systolic blood pressure (SBP) and diastolic blood pressure (DBP) were higher in the patients with hypertension, as shown in Table 1.

**Table 1:** Baseline characteristics of the study population

Characteristic	Patients	Healthy	*P* value
Age, mean (SD),y	61.43 (4.37)	56.33 (7.73)	0.299
Female (%)	68.80%	80.00%	0.291
BMI, mean (SD)	24.87 (4.95)	24.29 (0.71)	0.169
Pulse, mean (SD)	72.65 (7.57)	71.57 (7.07)	0.265
Blood sugar (mmol/L)	5.04 (0.30)	4.99 (0.42)	0.752
Total Cholesterol (mmol/L)	4.82 (0.55)	4.52 (0.41)	0.149
RBC count (× 10^12^/L)	4.48 (0.48)	4.49 (0.57)	0.958
WBC count (× 10^9^/L)	5.13 (0.93)	5.18 (1.41)	0.917
SBP, mean (SD) mmHg	144.69 (9.29)	113.60 (9.00)	< 0.0001
DBP, mean (SD) mmHg	88.61 (8.15)	75.14 (5.98)	< 0.0001

Demographic information and blood pressure indices. *BMI* body mass index, *RBC* red blood cell, *WBC* white blood cell, *SBP/DBP* systolic/diastolic blood pressure, *P* < 0.05 were considered statistically significant

### EA treatment reduced blood pressure indices compared with the baseline

After the six-week EA treatment, the blood pressure of patients differed significantly (Table 2). SBP (including daytime systolic blood pressure, dSBP and nighttime systolic blood pressure, nSBP) indices were significantly decreased more than 3 mmHg after EA treatment compared to that in the baseline (*P* < 0.001, *P* < 0.01, *P* < 0.05). While DBP and dDBP (daytime diastolic blood pressure) decreased significantly after EA treatment (*P* < 0.05, *P* < 0.05). nDBP (nighttime diastolic blood pressure) did not show any difference before and after treatment (*P* = 0.051).

**Table 2:** Outcome measurements during the study

Characteristic	Baseline	After treatment	MD (95% CI)	*P* value
SBP, mean (SD) mmHg	144.69 (9.29)	140.48 (9.77)	4.36 (1.97, 6.74)	< 0.001
DBP, mean (SD) mmHg	88.61 (8.15)	86.31 (8.02)	2.16 (0.45, 3.87)	< 0.05
dSBP, mean (SD) mmHg	147.32 (9.88)	143.02 (10.47)	4.48 (1.92, 7.04)	< 0.01
dDBP, mean (SD) mmHg	90.30 (8.49)	88.10.23 (8.24)	2.12 (0.38, 3.87)	< 0.05
nSBP, mean (SD) mmHg	136.20 (12.21)	132.59 (12.90)	3.77 (0.91, 6.64)	< 0.05
nDBP, mean (SD) mmHg	82.83 (8.78)	80.60 (9.64)	2.10 (-0.01, 4.21)	0.051

Blood pressure changes after six-week EA treatment. *MD* mean difference, *dSBP/dDBP* daytime systolic/diastolic blood pressure, *nSBP/nDBP* nighttime systolic/diastolic blood pressure. All data were analyzed by paired-sample *T* test, *P* < 0.05 were considered statistically significant

Alpha diversity showed no significant difference between Healthy group, BT group and AT group, whereas the three groups were clearly separated by PLS-DA.

In the current study, 73 samples were sequenced and a total of 3,440,986 high-quality raw sequences were generated. The average sequence length was 433 (min: 326, max: 458). Each sample was normalized to an equal sequencing depth and clustering. After filtering the low abundant (< 0.1%) OTUs, 870 OTUs were obtained at a 97% similarity level, from which 16 phyla, 30 classes, 55 orders, 98 families, 294 genera and 554 species were detected. Good's coverage was 99.82%, indicating the sequences identified in this study could represent the majority of bacterial sequences present in the samples. Although rarefaction curves of numbers of observed OTUs per sample suggested new bacteria would be expected with additional sequencing (Additional file 1: Fig. S1a), the rarefaction curves for the Shannon diversity index for each sample reached plateaus, indicating that the majority of the diversity was already procured (Additional file 1: Fig. S1b).

**Fig. 1:**
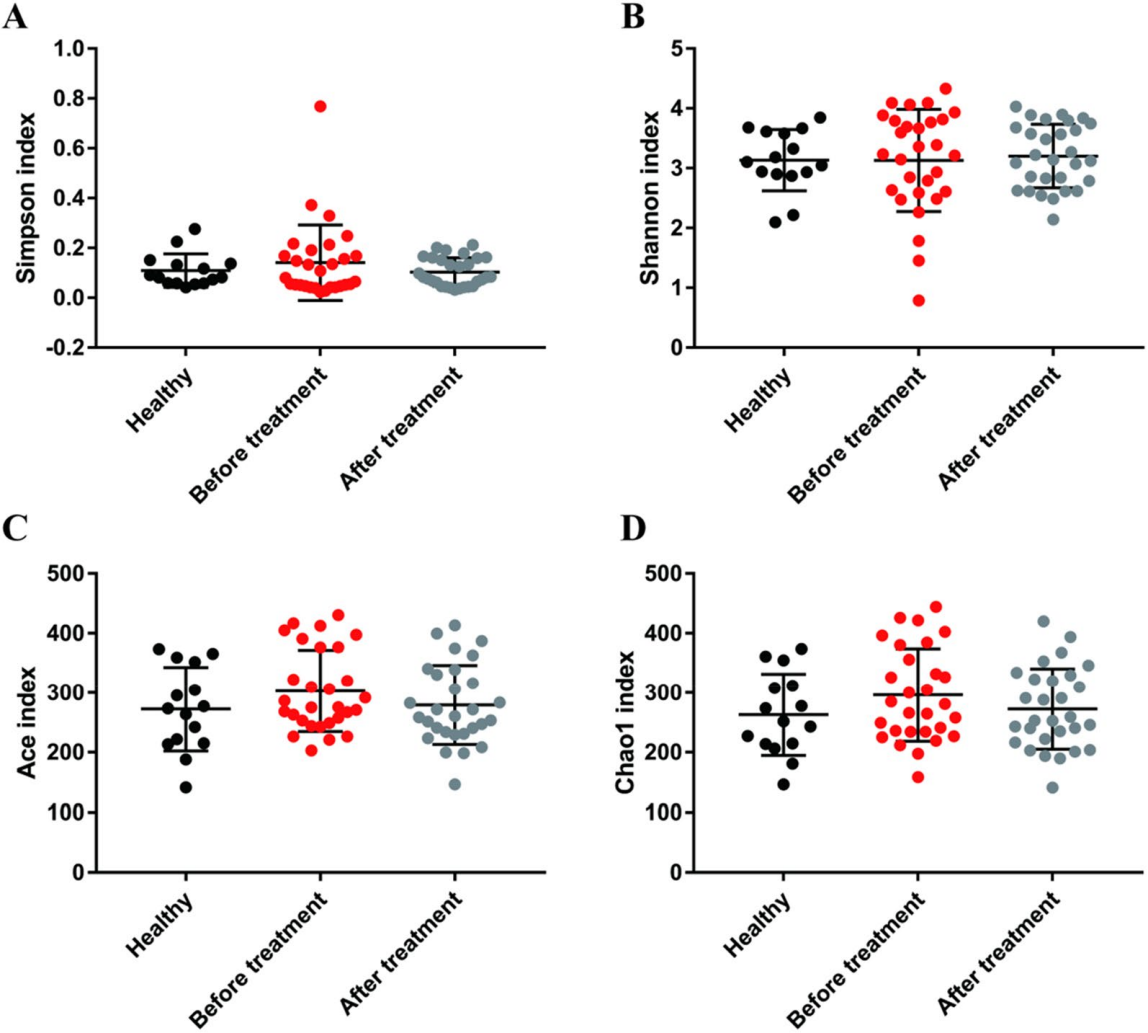
Influence of EA on the diversity and richness of gut microbiota. **a** Simpson and **b** Shannon index represent community diversity of OTUs. **c** Ace and **d** Chao1 index represent community richness of OTUs. There was no significant difference in Alpha diversity among three groups

**Fig. 2:**
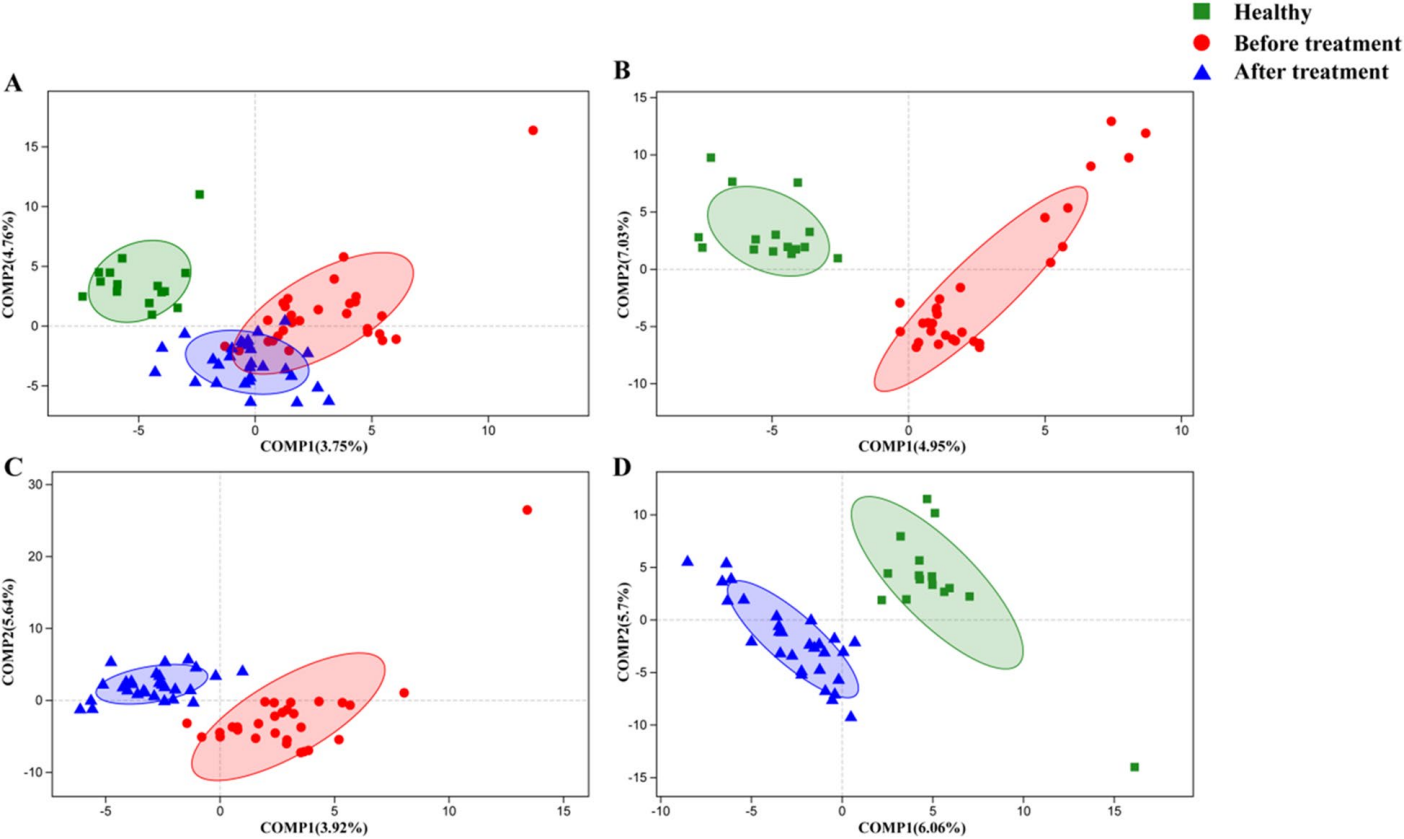
PLS-DA analysis for fecal microbiota of healthy group, before treatment group and after treatment group. **a** PLS-DA score plot for healthy group, before treatment group and after treatment group. **b** PLS-DA score plot for healthy group and before treatment group. **c** PLS-DA score plot for before treatment group and after treatment group. **d** PLS-DA score plot for healthy group and after treatment group. PLS-DA showed clear separation between Healthy and hypertension, and AT group showed a tendency toward the Healthy group

There was no difference in the alpha diversity respect determined by and Simpson/Shannon (species diversity) and Chao1/Ace (species richness) index between the Healthy group, BT group and AT group (Fig. 1). No difference among three groups was found in the beta diversity with unsupervised PCoA (Additional file 2: Fig. S2). However, supervised PLS-DA showed clear separation between Healthy and hypertension patients although the microbiome of AT group was similar to that of the BT group (Fig. 2).

### EA treatment improved intestinal microbiota structure in patients with stage 1 hypertension

**Fig. 3:**
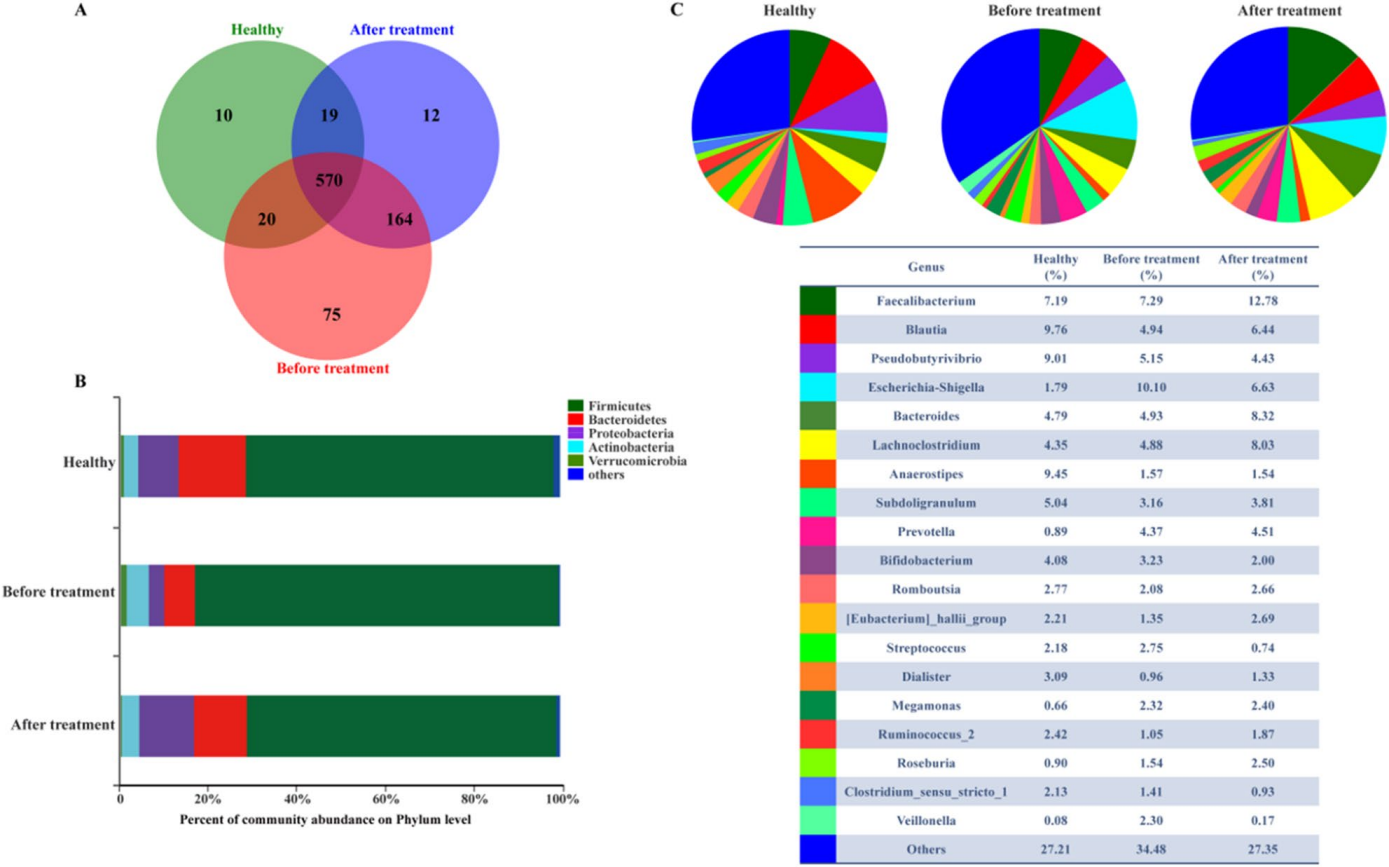
Modulation of the composition of gut microbiota by EA. **a** Venn diagrams demonstrate the number of OTUs shared between the Healthy, BT and AT group by the overlap. **b** Bar chart depicts the variability in phylum-level composition in each group. **c** Pie chart shows the proportion of reads in genus for the Healthy, BT and AT group

A Venn diagram was used to show the number of common and unique OTUs, thus intuitively describing sample similarity and overlap. The unique OTUs in the Healthy, BT, and AT groups were 10, 75 and 12 respectively. The common OTU number in three groups was 570, accounted for 92.08%, 68.75% and 74.51% of the total number of OTUs in the Healthy group (619), BT group (829) and AT group (765) (Fig. 3a). 5 phyla were identified with relative abundance more than 1%. The bacteria analysis showed that the relative abundance of predominant phyla in Healthy group, BT group and AT group varies widely although the species was similar (Fig. 3b). Figure 3c showed differences in the species and relative abundance of the dominant genus.

**Fig. 4:**
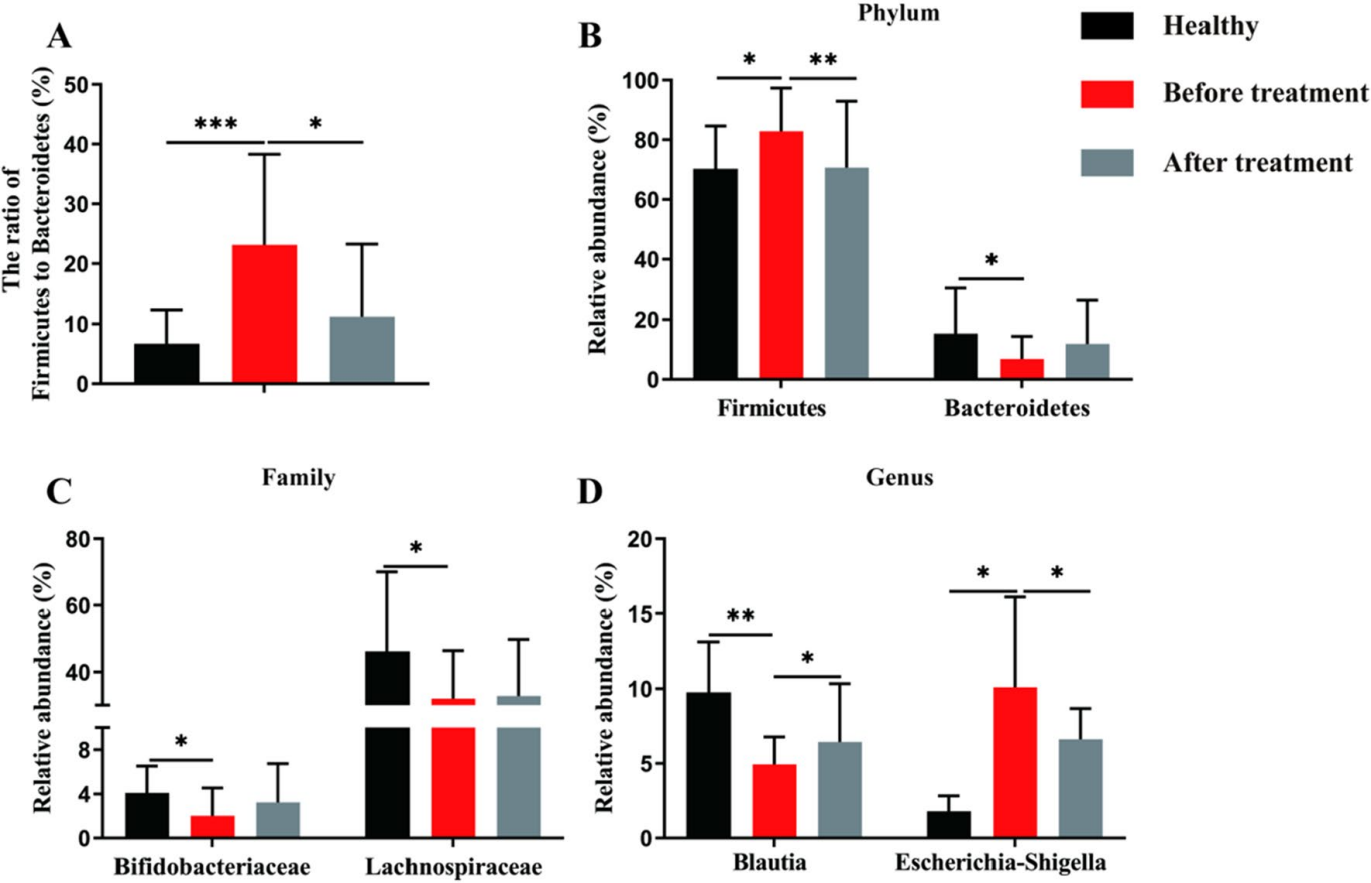
Influence of EA on the relative abundance of microbiota at each level. **a** The ratio of Firmicutes to Bacteroidetes ratio, and **b**–**d** differences at each taxonomic level (phylum, family and genus). All data were expressed as mean ± SD. *P < 0.05, **P < 0.01, ***P < 0.001

At the phylum level (Fig. 4b), the relative abundances of the dominant phyla such as *Firmicutes* and *Bacteroidetes* were showed significant dissimilarities among three groups. Higher abundance of *Firmicutes* (*P* < 0.05) and lower *Bacteroidetes* abundance (*P* < 0.05) were observed in the BT group compared to Healthy group. And EA treatment significantly decreased the relative abundance of *Firmicutes* (*P* < 0.01) compared to BT group. Moreover, compared to Healthy group, the ratio of *Firmicutes* to *Bacteroidetes* was significantly increased in the BT group (*P* < 0.001) and reduced in acupuncture group (*P* < 0.05) (Fig. 4a).

At the Family level (Fig. 4c), differences were observed in the *Bifidobacteriaceae* and *Lachnospiraceae*. *Bifidobacteriaceae* in BT group decreased compared to the Healthy group(*P* < 0.05). In addition, lower *Lachnospiraceae* abundance were observed in the Healthy group compared to the BT group (*P* < 0.05).

At the genus level (Fig. 4d), differences were observed in the *Blautia* and *Escherichia-Shigella*. The relative abundance of *Blautia* in the BT group was significantly decreased compared to the Healthy group (*P* < 0.01), but increased after EA treatment (*P* < 0.05). In contrast, BT group showed greater abundance of *Escherichia-Shigella* than that in the Healthy group (*P* < 0.05) and EA treatment (*P* < 0.05) decreased the abundance of *Escherichia-Shigella*.

### Potential correlation between clinical parameters and microbiota

**Fig. 5:**
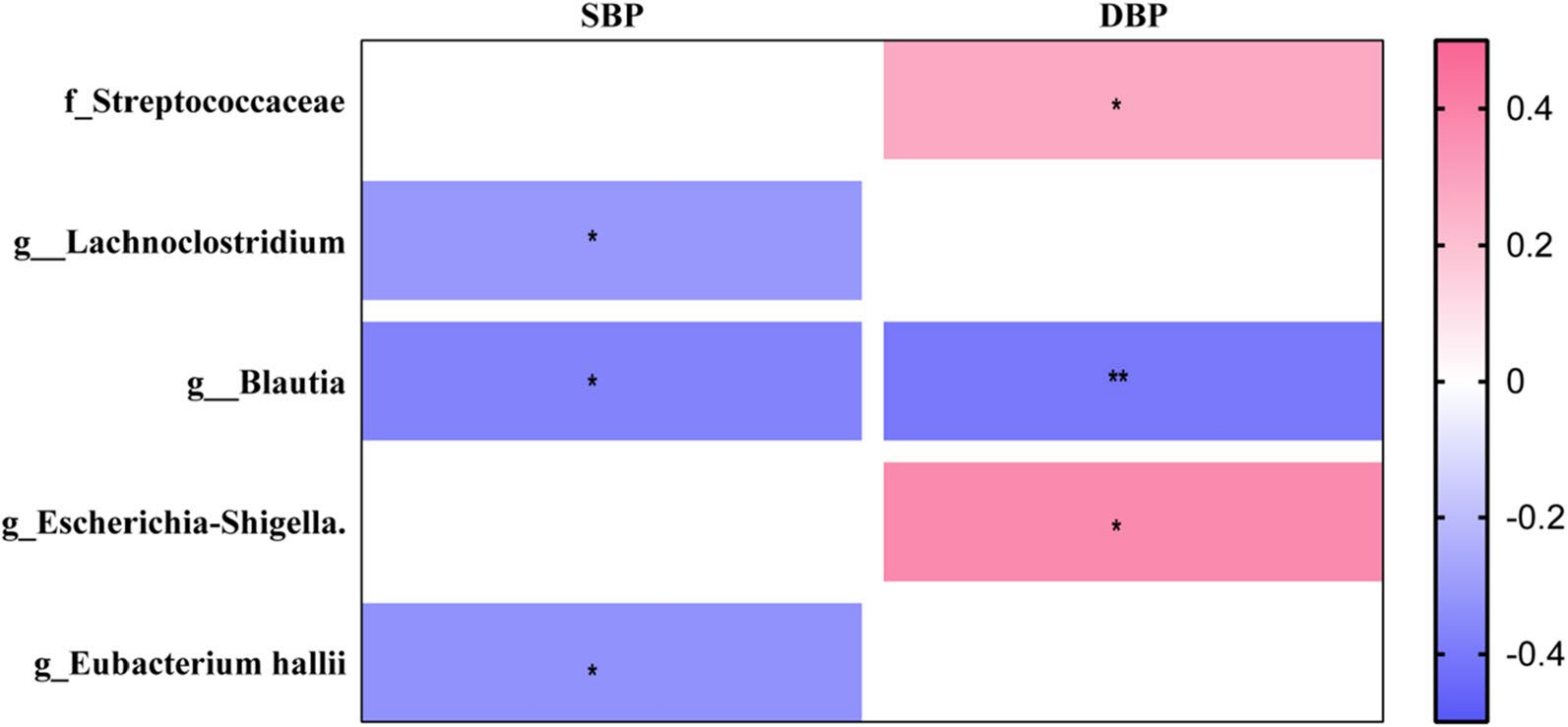
Spearman correlation plots between bacterial at family and genus level and BP values. R in different colors to show, the right side of the legend is the color range of different R values. *P < 0.05, **P < 0.01

We further assessed the correlation between the 10 most prevalent bacteria at family- and genus-level and blood pressure (Fig. 5). The *f_Streptococcaceae* (R = 0.27, *P* < 0.05) and *g_Escherichia-Shigella* (R = 0.37, *P* < 0.05) showed positive correlations with DBP. In addition, the negative correlations were observed for *g_Lachnoclostridium* (R = -− 0.31, *P* < 0.05) and *g_Eubacterium hallii* (R =  − 0.32, *P* < 0.05) with SBP. Moreover, g_*Blautia* were simultaneously found significant negative correlation with SBP (R =  − 0.37, *P* < 0.05) and DBP (R = − 0.40, *P* < 0.01).

## Discussion

There is convincing evidence suggesting that balance of intestinal microbiota is an important factor in hypertension although the mechanism is complex and in part unknown [21–23]. In this study, we investigated the antihypertensive effect of EA treatment in patients with stage 1 hypertension. The *Firmicutes* and *Bacteroidetes* changed in the patients before treatment, while EA significantly reduced *Firmicutes* abundance and the ratio of *Firmicutes* to *Bacteroidetes*. Moreover, EA increased the relative abundances of *Blautia* and reduced that of *Escherichia-Shigella* compared with BT group. Therefore, we suggest that the mechanism of reducing blood pressure by EA might be closely related with structural changes of gut flora.

This study showed that EA treatment at the aucpoints of taichong (LR3), taixi (KI3), renying (ST9) and neiguan (PC6) significantly decreased the SBP (− 4.4 mmHg; *P* < 0.001) and DBP (− 2.16 mmHg; *P* < 0.05). This finding was similar with a randomized, controlled, assessor-blinded pilot trial to assess the effects of acupuncture on prehypertension and stage 1 hypertension, in which SBP was reduced 5.7 mmHg [12]. Several high-quality evidences for patients with ungraded hypertension (SBP ≥ 140 mmHg or DBP ≥ 90 mmHg) also showed the approximate range of BP reduction fluctuated [24–27]. The reduction range of SBP and nDBP was 3.6–5.4 mmHg and 3–7 mmHg, respectively. Previous studies have estimated the extent of protection when BP is lowered. Girerd, et al. [28] showed that each 2 mmHg reduction in SBP was associated with a 25% reduction in stroke events. A reduction in SBP by at least 5 mm Hg in the general population was corresponding to a 9% reduction in mortality caused by coronary heart disease, and a 7% reduction in all-cause mortality [29, 30]. Therefore, EA has positive clinical meaningful effect on patients with stage 1 hypertension.

In addition, our data showed that EA decreased the ratio of *Firmicutes* to *Bacteroidetes*. In the healthy intestine, *Bacteroidetes* and *Firmicutes* contribute most types of the total bacterial species, and their ratio is often considered as a relative measurement of intestinal microbial health or dysbiosis [31]. The alteration of *Firmicutes*/*Bacteroidetes* ratio has been observed in multiple animal models of hypertension [22, 32, 33]. A pioneer study in the field compared the composition of microbiota between spontaneously hypertensive rats, chronic angiotensin II (Ang II) infusion rats and Wistar Kyoto (WKY) control rats. The results showed the *Firmicutes*/*Bacteroidetes* ratio in these models was 5 and 3 times that of the control group, respectively [21]. Same results were validated in spontaneously hypertensive stroke prone rats (SHRSP) [34]. In the experiment, SHRSP and WKY rats were performed fecal transplants after they were treated with broad-spectrum antibiotics. Ten weeks later, the WKY rats transplanted with caecum content from SHRSP showed significant increasing in the BP and *Firmicutes*/*Bacteroidetes* ratio, confirming the contribution of intestinal flora to hypertension. In the following years, similar results were obtained with others hypertensive animal models (i.e., obstructive sleep apnea-induced hypertensive rats; Dahl salt-sensitive and salt-resistant rats; deoxycorticosterone acetate (DOCA)-salt hypertensive mice) [35–37]. In our cohort, a significant higher abundance of *Firmicutes* and a lower abundance of *Bacteroidetes* were exhibited in individuals with stage 1 hypertension compared to healthy group. These findings were in agreement with the findings of Nosheen et al., who have demonstrated higher *Firmicutes*/*Bacteroidetes* ratio in patients with grade 3 hypertension [23]. Thus, the results indicated that intestinal dysbiosis in early stage promotes the development of hypertension, and EA may play a role in lowering blood pressure accompanying with improving gut dysbiosis.

Moreover, we observed the regulation of EA on *Blautia* and *Escherichia-Shigella* at genus level. *Blautia* is a group of bacteria that produces multiple SCFAs, including acetate [38], propionate [39], succinate and lactate in some subspecies [40]. SCFAs are a major class of bacterial metabolites and are widely recognized as potential mediators involved in regulating BP and inhibiting chronic inflammation [17, 41]. Acetate has been shown to reduce systolic and diastolic blood pressure, cardiac fibrosis and left ventricular hypertrophy in DOCA-salt hypertensive mice [37]. Another study reported that propionate reduced BP and attenuated Ang II-induced cardiac hypertrophy and fibrosis by mitigating systemic inflammation [17]. In addition, SCFAs may exert their effects by regulating renin release and vasomotor function via olfactory receptor and endothelial G protein-coupled receptor [42–44]. Lower abundance of *Blautia* has been detected in hypertensive patients with preeclampsia compared to healthy, pregnant control subjects [45], which was in agreement with our observation. The abundance of *Blautia* was also negatively correlated with systolic BP in spontaneously hypertensive rats and healthy male volunteers [46, 47]. In the present study, the significant increase of *Blautia* in EA group may be closely related to decreased blood pressure. *Escherichia-Shigella* is a group of gram-negative bacteria containing lipopolysaccharides (LPS) in their cell walls [22]. It is known that LPS activates toll-like receptor 4 (TLR4) pathway [48], which in turn induced the activation of proinflammatory cytokines (TNF-α, IL-6, and IL-1) and further led to inflammatory [49–51]. Bomfim and colleagues [22, 52] described that the inhibition of TLR4 activation by chronic treatment with anti-TLR4 antibody reduced both BP and endothelial dysfunction in spontaneously hypertensive rats. These results were in concordance with finding in Ang II-induced mice [53]. In this study, reduced level of *Escherichia-Shigella* after EA treatment may suggest an improvement inflammatory status of the host.

We also identified a significant correlation between BP values and 5 microbial taxa in the family at genus level. *F_Streptococcaceae*,*g_Eubacterium hallii* and *g_Lachnoclostridium* were observed correlation with blood pressure. Gut *Streptococcaceae* is also associated with inflammatory bowel disease [54] and liver cirrhosis [55]. Moreover, a study has showed that *Streptococcus* from gut might involve in the formation of atherosclerotic plaque microbiota [56]. *Eubacterium hallii* is a common member of the adult gut microbiota that can produce butyrate from lactate and acetate, and convert 1,2-propanediol to propionate [57, 58]. Udayappan, et al. [59] demonstrated that feeding *Eubacterium hallii* increased energy expenditure and fecal butyrate concentrations, thus modifying bile acid metabolism of db/db mice. *Lachnoclostridium*, a species belonging to *Lachnospiraceae*, was also a butyrate-producing bacterium [60], which can bind to metabolite-sensing G-protein coupled receptors (GPCRs) to regulate blood pressure via controlling gut homeostasis, host metabolism, and immune response [13].

In addition, we noticed an interesting phenomenon that hypertensive patients seem to produce more types of bacteria, although the abundance of these bacteria is very low. Further analysis showed that most of these bacteria belong to *Ruminococcaceae*, *Clostridiales* (Additional file 3: Table. S1). A phenome-wide association study has demonstrated the association between genus *Ruminococcus* and hypertension [61]. Another report shows that multiple species of *Clostridiales* (e.g., *Ruminococcaceae*, *Family_XIII*, etc.) were higher in non-treated hypertensive (SBP:153.1 ± 14.6) than normotensive (SBP:109.7 ± 7.1) [62]. An animal experiment also confirmed that high-carbohydrate, high-fat diet induced increasing *Clostridia* in hypertensive rats in the colonic microbiota [63]. Therefore, types of bacteria, especially *Ruminococcaceae* and *Clostridiales* should be enough attention in hypertensive patients.

As to the underlying mechanism or the target of acupuncture, neuroendocrine immunity system may be considered as an important pathway. As we mentioned, the association between intestinal microbiota and the host are complex and bi-direction. On the one hand, intestinal bacteria can modulate the host by producing a wide range of metabolites (SCFAs, bile acids, tryptophan et al.) and by their own component. Some of these metabolites and the bacterial components are risk markers for hypertension, inflammation and metabolic syndrome. On the other hand, the metabolism of host can regulate the microbiota in gut. For instance, the colonocyte metabolism works as a control switch, mediating a shift between homeostatic and dysbiosis communities [31]. Also, some of the metabolites such as butyrate activates PPARγ signaling in human epithelial cells [64] can drive the metabolism of surface colonocytes towards mitochondrial β-oxidation of fatty acids [65–67], in turn maintain epithelial hypoxia [68]. Therefore, it's hard to say which of these two aspects (the flora and the host) is the direct target of acupuncture. A recent review [69] summarized the mechanism of acupuncture in treating hypertension and found that renin–angiotensin–aldosterone system (RAAS), vascular endothelium, oxidative stress response, neuroendocrine system are all involved in the antihypertensive mechanisms of acupuncture while these mechanisms themselves are influenced by gut microbiota. In addition, previous studies showed EA could stimulate more SCFAs and improve the digestion. Therefore, further studies are needed to establish the contribution of specific bacteria controlled by EA to blood pressure regulation.

There were several limitations in this study. Firstly, we did not observe the long-term effect of EA on hypertension and intestinal microbiota. A study has reported that the antihypertensive effect of acupuncture could be decreased with the elapse of time [25]. Another limitation is that the gut microbial community would be sensitive to environmental factors [70], larger cohort containing multi-types of hypertensive patients are needed for further investigation.

## Conclusion

The present study shows that electroacupuncture can effectively lower blood pressure and improve the structure of intestinal microbiota which are correlate with the alteration of blood pressure by EA. These findings could inspire future research for understanding the mechanism underlying the therapeutic effects of acupuncture on hypertension.

## Supplementary Information

**Additional file 1: Fig. S1** Alpha diversity rarefaction curves of samples based on species-level OTUs.

**Additional file 2: Fig. S2** Analysis of principal coordinate analysis (PCoA) with the bray_curtis distance matrix on the OTU level.

**Additional file 3: Table S1. **Unique OTUs in BT group.

## Data Availability

The datasets used and/or analysed during the current study are available from the corresponding author on reasonable request.

## Abbreviations

EA: Electroacupuncture
BT: Before treatment
AT: After treatment
CVD: Cardiovascular diseases
BP: Blood pressure
SCFAs: Short-chain fatty acids
Ang II: Angiotensin II
WKY: Wistar Kyoto rat
SHRSP: Spontaneously hypertensive stroke prone rats
LPS: Lipopolysaccharides
TLR4: Toll-like receptor 4
GPCRs: G-protein coupled receptors
PVN: Paraventricular nucleus
ROS: Reactive oxygen species
